# Effects of a Synchronous Telehealth Exercise Program on Clinical, Functional, and Psychosocial Outcomes in Individuals with Type 2 Diabetes Mellitus (RED Study): A Randomized Clinical Trial

**DOI:** 10.3390/ijerph23060773

**Published:** 2026-06-08

**Authors:** Samara Nickel Rodrigues, Bruno Veiga Guterres, Maurício Tatsch Ximenes Carvalho, Rodrigo Sudatti Delevatti, Cristine Lima Alberton

**Affiliations:** 1 School of Physical Education and Physiotherapy, Federal University of Pelotas, Pelotas 96055-630, RS, Brazil; samara-nrodrigues@hotmail.com (S.N.R.); bveigaguterres@gmail.com (B.V.G.); carvalhomauricio960@gmail.com (M.T.X.C.); 2 Department of Physical Education, Center of Sports, Federal University of Santa Catarina, Florianopolis 88040-900, SC, Brazil; rodrigo.delevatti@ufsc.br

**Keywords:** eHealth, cardiometabolic risk, glycemic control, physical function, sleep quality

## Abstract

**Highlights:**

**Public health relevance—How does this work relate to a public health issue?**

- Individuals with type 2 diabetes mellitus are at increased risk of cardiovascular diseases and commonly face barriers to participating in face-to-face exercise programs.
- Accessible and scalable exercise delivery strategies, such as synchronous telehealth programs, may help overcome barriers to physical activity participation among high-risk populations.

**Public health significance—Why is this work of significance to public health?**

- This randomized clinical trial demonstrated that a synchronous telehealth exercise program improved functional performance and sleep quality in individuals with type 2 diabetes mellitus.
- The intervention also promoted acute reductions in post-session capillary blood glucose levels, supporting the potential role of telehealth-based exercise in cardiometabolic risk management.

**Public health implications—What are the key implications or messages for practitioners, policy makers and/or researchers in public health?**

- Synchronous telehealth exercise programs may represent a feasible and accessible strategy to promote functional benefits and improve self-reported sleep quality in individuals with type 2 diabetes mellitus.
- The findings support the integration of telehealth exercise interventions into public health strategies aimed at expanding access to physical activity programs and improving cardiometabolic health in populations at risk of cardiovascular disease.

**Abstract:**

Individuals with type 2 diabetes mellitus (T2D) are at increased cardiovascular risk. Although exercise is an important strategy for reducing cardiometabolic risk, accessible and scalable intervention delivery strategies, such as synchronous telehealth programs, remain underexplored. This randomized clinical trial (RED Study; NCT05362071) investigated the effects of a 12-week synchronous telehealth exercise program on clinical, functional, and psychosocial outcomes in adults with T2D. Thirty-three participants (55.8 ± 10.1 years) were randomized to an intervention group (INT; *n* = 17), which performed supervised combined aerobic and resistance exercise via video calls (2–3 sessions/week), or a control group (CON; *n* = 16). Glycated hemoglobin (HbA1c) was the primary outcome. Secondary outcomes included capillary blood glucose, blood pressure, functional performance, and psychosocial parameters. Assessments were conducted at baseline and post-intervention by blinded evaluators, and analyses were conducted using linear mixed-effects models in an intention-to-treat analysis. No significant interaction effect was observed for HbA1c (*p* > 0.05). However, significant group × time interactions favored the INT for functional performance outcomes, including the 30 s Chair Stand (*p* = 0.02), Arm Curl (*p* < 0.001), Timed Up and Go (*p* = 0.01), and 2-Minute Step Test (*p* = 0.01), as well as sleep quality (*p* < 0.001). Depressive symptoms decreased over time (*p* = 0.03) in both groups. Additionally, the INT showed reductions in post-session capillary blood glucose across mesocycles 1, 2, and 4 (*p* = 0.03). The synchronous telehealth exercise program was not superior to the control condition in reducing HbA1c; however, it improved functional performance, enhanced sleep quality, and promoted acute reductions in glycemic levels in individuals with T2D.

## 1. Introduction

Type 2 diabetes mellitus (T2D) is a condition associated with multiple complications that can significantly impact patients’ physical function and quality of life. The spectrum of diabetes-related complications includes micro- and macrovascular complications, impairments in the musculoskeletal and digestive systems, as well as deficits in cognitive function and mental health [1]. Individuals with T2D present neuromuscular alterations, such as loss of motor units, reduced rate of force development, and impaired motor control, particularly in the presence of peripheral neuropathy [2]. These changes compromise functional performance and postural stability, increasing the risk of falls [2] and hip fractures, the lifetime risk of which is 40–70% higher in individuals with T2D compared to those without the disease [3].

In addition, psychological comorbidities are common among individuals with T2D. It is estimated that one in five adults with T2D experiences depression, a mental disorder characterized by persistent sadness, low mood, and loss of interest or pleasure [4,5]. Approximately one-third of these individuals also experience diabetes-related emotional distress (ED), characterized by feelings of guilt, fear, and frustration associated with self-care demands, concerns about future complications, and challenges in disease management. This condition may present similarly to mild depressive symptoms and may potentially progress to depression [4,5].

Another relevant aspect is the relationship between T2D and sleep quality (SQ). Individuals with T2D frequently experience sleep disorders, with reported prevalence ranging from 24 to 86% for obstructive sleep apnea, approximately 39% for insomnia, and from 8 to 45% for restless legs syndrome [6]. These estimates are derived from heterogeneous evidence and may vary depending on study design and diagnostic criteria. Alterations in sleep duration and variability, particularly sleeping less than 6 h or more than 9 h per night, are associated with poorer glycemic control [7].

Therefore, individuals with T2D face complex and multifaceted challenges in integrating disease management into their daily lives. Psychosocial well-being is a critical component of treatment and self-management in T2D [6]. Psychological and social factors may interfere with an individual’s ability to perform disease management tasks and negatively affect overall health status [6].

In this context, regular physical exercise stands out as an effective strategy for T2D management. However, many individuals face barriers to adhering to exercise recommendations due to comorbidities such as obesity and depression, as well as diabetes-related complications, such as neuropathy, which often limit the ability to engage in regular physical activity [8]. Additionally, factors such as lack of time and motivation are commonly reported as barriers to regular exercise [9].

The adoption of telehealth services, including remote exercise interventions, has become even more relevant following the COVID-19 pandemic [10,11]. Among these alternatives, telehealth exercise programs have gained particular attention. These interventions use digital platforms to provide remote professional guidance, enabling individuals with T2D to engage in exercise safely, either synchronously or asynchronously, in their home environment [11,12]. Synchronous telehealth exercise interventions have shown promising results in improving health outcomes across various chronic conditions [10,11]. In individuals with T2D, synchronous telehealth exercise interventions have been associated with significant benefits, including improvements in glycemic control, reductions in body weight, and enhanced psychosocial outcomes [13,14,15].

Despite advances in the field, evidence regarding synchronous supervised telehealth exercise interventions remains limited. This type of intervention has the potential to enhance exercise safety and provide immediate feedback on movement execution. In addition, this approach has emerged as a strategy to overcome barriers associated with in-person training, thereby expanding access to exercise participation and health care support for this population. Moreover, most previous interventions have been conducted over relatively short durations, with limited investigation of acute glycemic responses and psychosocial outcomes such as SQ and ED. Therefore, this study aimed to investigate the effects of a synchronous telehealth exercise program on clinical, functional, and psychosocial outcomes in individuals with T2D.

## 2. Materials and Methods

### 2.1. Study Design

This study was designed as a single-center, randomized, controlled, superiority trial with two parallel arms and a 1:1 allocation ratio. Outcome assessors and data analysts were blinded to group assignment. Participants were randomized to an intervention group (INT), which participated in a synchronous telehealth exercise program, or to a control group (CON). The study was approved by the Research Ethics Committee of the School of Physical Education and Physiotherapy at the Federal University of Pelotas (CAAE: 55791622.8.0000.5313). The study was prospectively registered at ClinicalTrials.gov (NCT05362071), and the study protocol has been published elsewhere [16]. The study was conducted according to the registered protocol and reported in accordance with the Consolidated Standards of Reporting Trials (CONSORT) guidelines [17].

### 2.2. Randomization

The randomization sequence was stratified by sex and duration of T2D diagnosis (<5 years or ≥5 years) and generated in blocks of varying sizes (4 or 8) with a 1:1 allocation ratio using the RAND function in Microsoft Excel. Allocation concealment was ensured by an independent researcher who generated the allocation sequence and was not involved in participant recruitment, assessments, or intervention delivery.

### 2.3. Participants

The study participants comprise male and female patients with T2D from the city of Pelotas, located in the state of Rio Grande do Sul in the southern region of Brazil. The inclusion criteria were: 1. Being under medical treatment using oral hypoglycemic agents; 2. Aged ≥45 years old; 3. Not involved with physical exercises for at least three months (the regular practice of exercise was defined as performing any modality of physical training for at least 20 min on two or more days of the week); and 4. Sufficient literacy skills to independently complete the self-administered questionnaires. The exclusion criteria were: 1. Insulin prescription and use; 2. History of cardiovascular disease (except drug-controlled high blood pressure); 3. Presence of severe autonomic neuropathy, severe peripheral neuropathy, or history of foot injuries, proliferative diabetic retinopathy, severe non-proliferative diabetic retinopathy; 4. Muscle or joint impairment that precludes performing physical exercises safely; and 5. Lack of internet access.

### 2.4. Sample Size Calculation

The sample size calculation was performed using G*Power software (version 3.9.1.4) for F tests, based on the primary outcome of HbA1c. Mean and standard deviation values were extracted from Akinci et al. [18]. An effect size of *f* = 0.24, a significance level of α = 0.05, and a statistical power of 80% were assumed. The calculation indicated a total sample size of 38 participants to be randomized between the two groups.

### 2.5. Recruitment

Volunteers included in the sample were recruited through advertisements on social media and at Basic Health Units (BHUs) across five health districts in the city of Pelotas. BHUs serve as the primary entry point to the Brazilian Unified Health System (SUS) and provide free multidisciplinary care. Recruitment took place between May 2022 and June 2023 and was conducted in three intervention waves. Initially, interested individuals were contacted by telephone, during which they received detailed information about the study objectives and completed a questionnaire to assess eligibility. Eligible participants were then invited to take part in the study and were instructed regarding the subsequent procedures.

### 2.6. Procedures

After written informed consent was obtained in person, all baseline assessments were scheduled. Clinical, functional, and anthropometric outcomes were assessed in person, whereas the remaining outcomes were collected through self-administered questionnaires delivered online via Google Docs at baseline (week 0) and post-intervention (week 13). To clarify any doubts regarding the responses, participants received explanatory instructions at the beginning of the questionnaire, and follow-up phone calls were conducted before and immediately after its completion. In addition, the assessor was available to provide procedural support during questionnaire completion, when necessary, without influencing participants’ responses.

All assessors were blinded to group allocation and were not involved in any other stage of the study. Due to the nature of the intervention, participants could not be blinded to group assignment. After completing baseline assessments, participants were randomized to the INT, which participated in a remotely supervised home-based exercise program lasting 12 weeks, or to the CON.

Additionally, only participants in the INT underwent acute capillary blood glucose measurements before and immediately after a session during the first week of mesocycles 1, 2, and 4. Therefore, these analyses should be interpreted as exploratory within-intervention outcomes.

### 2.7. Intervention and Control Procedures

#### 2.7.1. Intervention Group

The sessions were conducted via video calls using WhatsApp (version 2.26), with a maximum of five participants per session to ensure adequate supervision. The intervention was delivered twice per week on non-consecutive days during the first six weeks and three times per week on non-consecutive days during the subsequent six weeks. All sessions within each recruitment wave were conducted by the same instructor, who supervised participants throughout the intervention period, ensuring consistent delivery.

The program was structured into four mesocycles, each lasting three weeks. The overall session structure was consistent across mesocycles and adjusted in the subsequent mesocycle according to the planned progression in volume and/or intensity. Each session consisted of a 5 min warm-up involving joint mobility exercises, 37 to 57 min of the main exercise phase (depending on the mesocycle), which involved combined training (resistance and aerobic exercises), and a final 5 min cool-down period with stretching exercises.

The main phase was organized into three blocks. Blocks 1 and 2 included three resistance exercises performed using body weight and standardized alternative materials (a pair of 500 mL plastic bottles filled with sand), as well as one stationary aerobic exercise. Between blocks 1 and 2 and again during block 3, participants performed free walking within the available space in their home environment as a complementary aerobic activity. These walking periods were not continuously visually supervised due to the nature of home-based execution. However, brief interruptions in visual contact occurred only when participants moved outside the camera frame. In such cases, verbal communication was used to promptly re-establish monitoring and ensure continuity and safety of the session.

Aerobic exercise intensity was prescribed based on the Borg 6–20 Rating of Perceived Exertion (RPE) scale [19]. Resistance exercises were performed at either usual or maximal movement speed. Usual movement speed was defined as the self-selected pace adopted by participants during exercise execution, whereas maximal movement speed was defined as the fastest safe speed that could be performed while maintaining correct movement technique. Exercise duration and adherence were recorded for each session, and intensity was guided using standardized instructions combined with RPE monitoring.

The alternative materials were delivered to participants during a home visit prior to the intervention beginning. Participants were also instructed not to engage in any additional exercise-related activities during the study period. Throughout the intervention, participants were monitored in real time during exercise sessions through video calls, allowing instructors to provide immediate feedback regarding exercise execution and safety. At the beginning of each training session, participants were systematically screened for signs and symptoms potentially related to adverse events, including hypoglycemia and hypotension. Any adverse events reported during the intervention period were monitored and recorded. In the event of medical complications, participants would be instructed to interrupt the session immediately, and emergency support procedures would be available as needed. The full exercise program periodization is presented in Table 1.

#### 2.7.2. Control Group

Participants allocated to the CON received recommendations for physical activity based on chapters of the Physical Activity Guide for the Brazilian Population (2021) [20]. After completing all baseline assessments, CON participants received materials via WhatsApp corresponding to chapters 1, 4, and 5, which address the following topics: “Understanding Physical Activity,” “Physical Activity for Adults,” and “Physical Activity for Older Adults.” When delivery via WhatsApp was not possible, a printed booklet was provided to the participant. At the end of the 12-week intervention period, the same booklet was made available to participants allocated to the INT, along with an explanation of its content.

### 2.8. Outcomes

All outcomes were assessed in a blinded manner at two time points: baseline (week 0) and post-intervention (week 13). Clinical outcomes included HbA1c and blood pressure (BP). Functional outcomes included lower-limb muscular endurance, upper-limb muscular endurance, agility and dynamic balance, lower-limb flexibility, and aerobic capacity. Psychosocial outcomes included quality of life (QOL), sleep quality (SQ), ED, and depressive symptoms (DS).

In addition, other outcomes were collected, including anthropometric measures (body mass, height, and waist circumference), dietary habits, and physical activity level. Outcomes were measured for all randomized participants, regardless of adherence or completion status. Adherence, capillary blood glucose during the session, and subjective well-being perception were assessed only in participants from the INT who completed the intervention.

Participants who withdrew from the study at any time after randomization were invited to complete the final assessments. Final assessments were conducted at least 48 to 72 h after the last program session to ensure that acute residual effects did not influence chronic outcome measures.

#### 2.8.1. Primary Outcome

##### Glycated Hemoglobin (HbA1c)

Venous blood samples were collected in an outpatient setting by trained professionals. Samples were processed and stored according to standard laboratory procedures for subsequent analysis. HbA1c was measured using high-performance liquid chromatography (HPLC) and expressed as a percentage (%).

Participants were informed that fasting was not required for the test and were instructed to maintain their usual medication regimen, including oral antidiabetic drugs, unless otherwise advised by their physician. The test was scheduled in advance with each participant, who was required to attend the laboratory on the scheduled date and time for blood collection. In cases where adverse circumstances prevented laboratory collection, a home visit would be arranged, in which a laboratory professional would collect the blood sample at the participant’s residence; however, this did not occur in the included sample.

#### 2.8.2. Secondary Outcomes

##### Blood Pressure

Systolic (SBP) and diastolic (DBP) blood pressure measurements in the office were obtained using an automatic blood pressure monitor (HEM-7320, OMRON, Hong Kong, China). The participant was kept at rest for 5 min, after which, one measurement was performed on each arm. Two additional measurements were then obtained on the arm with the highest value, always with a 1 min interval between measurements. The mean of the repeated measurements was used for analysis. Appropriate cuff sizes were selected according to participant characteristics. Assessments were conducted at standardized times of day, and participants were instructed to avoid strenuous physical activity for 24 h before evaluation, maintain their usual medication, sleep, and dietary routines, and avoid caffeine-containing beverages and alcohol on the day of assessment.

##### Functional Performance

The 30 s Chair-Stand test was used to assess lower-limb strength. Participants were instructed to sit and stand from a 42 cm-high chair (seat height), without using their upper limbs, as many times as possible within 30 s [21].

The Arm Curl test was performed to assess upper-body strength. The test was conducted using a dumbbell held in the dominant hand, with a load of 2 kg for women and 4 kg for men. Participants were instructed to perform the maximum number of elbow flexion repetitions through the full range of motion within 30 s [21].

The Timed Up and Go (TUG) test was used to measure agility and dynamic balance. The test began with the participant seated in a chair, with a cone positioned 3 m in front of them. Participants were instructed to stand up, walk as quickly as possible without running, go around the cone, and return to the starting position. The shortest time of the two attempts was recorded as the test result. In addition, the test was also performed at the usual walking speed [22].

The Sit-and-Reach Flexibility Test was performed using the Wells bench to assess lower limb flexibility. Participants were barefoot and seated facing the base of the box, with legs extended and together. One hand was placed over the other, and the arms were raised vertically. At the evaluator’s signal, the participant bent the trunk forward and reached as far as possible along the graduated ruler without bending the knees or performing bouncing movements. Two trials were performed, and the highest value was recorded [23].

The 2 min Step Test was conducted to estimate aerobic capacity. The test measured the maximum number of knee raises performed within 2 min. At the signal, the participant began stationary marching (without running) and performed as many knee raises as possible during the 2 min period. The minimum knee height was defined as the midpoint between the patella and the anterior superior iliac spine. The evaluator counted the number of right knee raises [21].

##### Quality of Life (QOL)

QOL was assessed using the European Health Interview Survey–Quality of Life 8-Item Index (EUROHIS-QOL 8-Item), an instrument validated for the Brazilian population [24]. The questionnaire consisted of eight items addressing general QOL, general health, energy, activities of daily living, self-esteem, social relationships, financial resources, and home environment. Each item was answered individually using a five-point Likert scale, ranging from “very bad” to “very good” (evaluation scale), “very dissatisfied” to “very satisfied” (satisfaction scale), and “not at all” to “extremely” (intensity scale). The total score ranged from 8 to 40 points, with higher scores indicating better perceived QOL.

##### Sleep Quality (SQ)

SQ was assessed using the Pittsburgh Sleep Quality Index (PSQI), validated for the Brazilian population [25]. The questionnaire included 19 self-rated questions and five additional questions answered by roommates when applicable. If the participant did not have a roommate, these additional questions were not completed and were not included in the scoring. The items were grouped into seven components, each scored from 0 to 3, with higher scores indicating worse sleep quality.

##### Depressive Symptoms (DS)

DS were assessed using the Patient Health Questionnaire-9 (PHQ-9), validated for the Brazilian population [26]. The instrument evaluates the presence of depressive symptoms over the previous two weeks using a four-point Likert scale (0–3). It consists of nine items with response options ranging from “not at all” (0 points) to “nearly every day” (3 points). An additional tenth question assesses the impact of symptoms on daily functioning. Total scores ranged from 0 to 27, with lower scores indicating fewer depressive symptoms.

##### Diabetes-Related Emotional Distress (ED)

ED was assessed using the Brazilian version of the Problem Areas in Diabetes Scale (B-PAID) [27]. The instrument evaluates diabetes-related emotional distress and the impact of diabetes and its treatment on participants’ lives. Responses were provided on a five-point Likert scale ranging from “no problem” (0) to “serious problem” (4). The total score ranged from 0 to 100 and was calculated by summing the responses to the 20 items (0–4) and multiplying the total by 1.25. Higher scores indicated greater emotional distress.

#### 2.8.3. Intervention-Only Outcomes

##### Adherence

Adherence to the program was determined by the number of sessions completed relative to the total prescribed during the intervention. Total adherence (%) was calculated as the ratio of completed sessions to prescribed sessions. Additionally, adherence was analyzed by mesocycle, considering the proportion of sessions completed within each specific period of the program. Weekly absolute frequency was calculated as the average number of sessions completed per week across mesocycles. Results were expressed as mean and standard deviation (SD) for the total number of sessions, relative frequency (%), and absolute frequency (sessions/week), and were analyzed using descriptive statistics.

##### Capillary Blood Glucose During the Session

Capillary blood glucose was measured before and immediately after an exercise session at the beginning of mesocycles 1, 2, and 4, only among participants in the INT who completed the intervention. Measurements were not performed at the beginning of mesocycle 3, as exercise sessions were identical to those of mesocycle 2, with progression occurring only in weekly frequency. Measurements were obtained from blood samples (0.6 μL) collected from the fingertip using disposable lancets and test strips, with immediate analysis using a portable glucometer (Accu-Chek Guide, Roche, São Paulo, Brazil), with an approximate reading time of 4 s.

To minimize contact between participants and researchers during the data collection period [28], due to the restrictive measures in place, participants were instructed to perform their own blood glucose measurements, a procedure they were already familiar with, following standardized instructions. All necessary materials, as well as instructions and the recording schedule, were provided during the first visit. Session times and pre-session glucose assessment procedures were standardized throughout the intervention. However, meal intake and medication timing were not strictly controlled, and participants received only general recommendations regarding these factors.

##### Subjective Well-Being Perception

Subjective well-being perception was assessed using the question: “In your opinion, how much did participation in the program improve your overall sense of well-being?”, applied only to participants in the INT at the end of the intervention. Responses were structured on a 5-point Likert scale, ranging from “dissatisfied” to “very satisfied”. The outcome was self-administered and collected online via Google Forms.

#### 2.8.4. Other Outcomes

##### Anthropometric Assessment

Body mass and height were measured using a digital scale (HN-289, OMRON, China) and a compact stadiometer (MD, São Paulo, Brazil), respectively. Body mass index (BMI) was calculated using the equation: BMI = body mass (kg)/height^2^ (m). Waist circumference was measured at the midpoint between the iliac crest and the last rib. From these measurements, the waist-to-height ratio was calculated.

##### Physical Activity Levels

Physical activity level was assessed using the short version of the International Physical Activity Questionnaire (IPAQ-C), validated for the Brazilian population [29]. The questionnaire consisted of eight self-administered items assessing walking, moderate- and vigorous-intensity physical activity, and sitting time during the previous seven days. Data were expressed in minutes, and the metabolic equivalent was calculated (1 MET: 3.5 mL/kg/min). As with the other questionnaires, the instrument was administered electronically, as previously described [30]. Participants were categorized according to IPAQ classification criteria as very active, active, or insufficiently active. Participants were classified as very active if they performed vigorous-intensity physical activity on ≥5 days/week for ≥30 min/session or vigorous-intensity activity on ≥3 days/week for ≥20 min/session combined with moderate-intensity activity and/or walking on ≥5 days/week for ≥30 min/session. Participants were classified as active if they performed vigorous-intensity activity on ≥3 days/week for ≥20 min/session, moderate-intensity activity and/or walking on ≥5 days/week for ≥30 min/session, or any combination of physical activities totaling ≥150 min/week over ≥5 days/week. Participants who performed some physical activity but did not meet the criteria above, as well as those who did not perform any physical activity, were classified as insufficiently active.

##### Eating Habits

Eating habits were assessed using a Food Frequency Questionnaire (FFQ), as previously applied in other studies [31]. The questionnaire consisted of a list of 16 food items derived from previous research [32,33]. According to the Brazilian Dietary Guidelines, foods were classified into two groups: in natura/minimally processed foods and processed/ultra-processed foods. Scores were generated for each group based on the reported frequency of consumption. Scores ranged from 0 to 32 points for each group, with higher scores indicating healthier eating habits. A global healthy eating score was also calculated from the sum of all evaluated foods, ranging from 0 to 64 points, with higher scores indicating better dietary patterns.

### 2.9. Statistical Analysis

Data were organized and analyzed using SPSS software (version 27.0.1) (IBM Corp., Armonk, NY, USA). Descriptive statistics were used to characterize the sample, with results presented as mean ± standard deviation or absolute frequency.

Analyses were conducted according to the intention-to-treat principle. For continuous outcomes, linear mixed models were used, with participants treated as a random effect. Time (pre- and post-intervention) and group (intervention and control), as well as the group × time interaction, were included as fixed effects when applicable. Missing data were assumed to be missing at random (MAR), as withdrawals appeared to be primarily related to adherence difficulties and loss of interest rather than outcome values themselves. For outcomes assessed only in the intervention group, time was included as a fixed effect.

A compound symmetry covariance structure was adopted. This structure was selected a priori for its simplicity, suitability for small sample repeated-measures designs, and model convergence. Alternative covariance structures were not formally compared, and model fit indices (AIC/BIC) were not used for model selection. The models included random intercepts for participants only, with no random slopes specified. Missing data were handled by the mixed-model approach, which uses all available observations under the assumption that the data are missing at random. Post hoc comparisons were performed using preplanned pairwise contrasts based on estimated marginal means. Results are presented as mean, standard deviation, and 95% confidence interval (95% CI). The level of significance was set at 5% (*p* < 0.05).

Between-group effect sizes were calculated for outcomes with significant results using Cohen’s *d* [34], based on change scores (post minus baseline) and standardized by the pooled baseline standard deviation [35], and were classified as small (≥0.2), moderate (≥0.5), or large (≥0.8). For repeated-measures analyses, effect size was estimated from the mean change between pre- and post-intervention assessments and standardized by the mean standard deviation of both assessments, following practical recommendations for this design [36,37].

## 3. Results

### 3.1. Participants

A total of 104 telephone contacts were made through voluntary recruitment. Of these, 21 individuals lost interest in participating, and 36 did not meet the eligibility criteria. The main reasons for ineligibility were insulin use (*n* = 15), history of cardiovascular disease (*n* = 7), regular engagement in physical exercise (*n* = 6), physical limitations (*n* = 2), diagnosis of prediabetes (*n* = 2), age below 45 years (*n* = 2), and residence in another city (*n* = 2).

Thus, 47 individuals were considered eligible. However, 14 withdrew before completing the baseline assessments. Therefore, 33 participants completed the baseline assessments and were subsequently randomized to the INT (*n* = 17) or CON (*n* = 16) group. This final sample size was lower than the number originally estimated in the study protocol. Difficulty in reaching the planned number of participants was likely influenced by the COVID-19 pandemic context during which recruitment was conducted, as well as by the study’s eligibility criteria.

**Figure 1:**
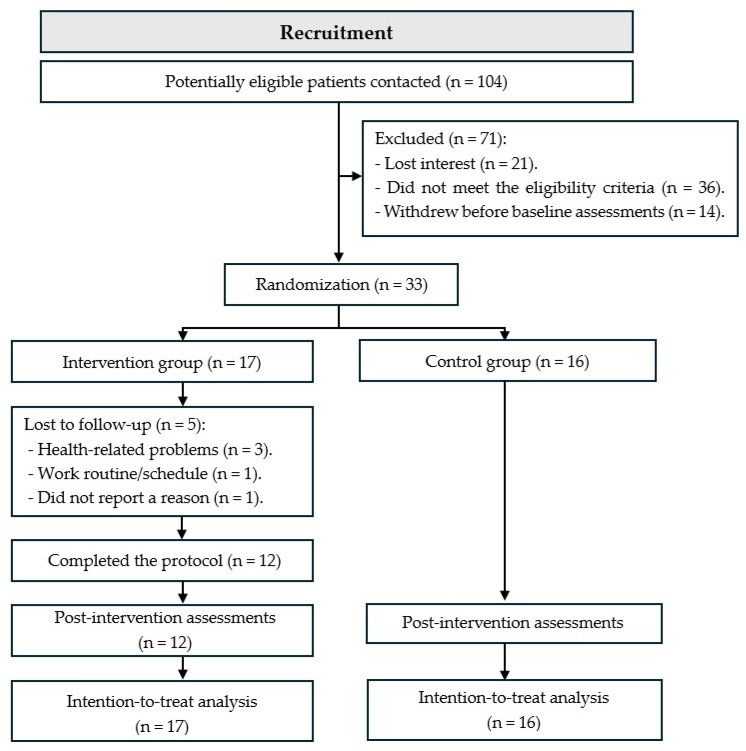
Flow diagram of participants through the study.

During the study, 5 participants in the INT discontinued the intervention, and 1 was unable to complete all post-intervention assessments. Thus, post-intervention assessments were completed by 12 participants in the INT and 16 in the CON. All 33 randomized participants were included in the intention-to-treat (ITT) analysis, comprising 17 participants in the INT and 16 in the CON. No adverse events potentially related to the study were reported. Figure 1 presents the participant flowchart.

### 3.2. Sample Characteristics

The characteristics of the 33 participants are presented in Table 2. Baseline characteristics of completers and non-completers within the intervention group are presented in Supplementary Table S1. Overall, both groups showed similar baseline characteristics, including age, anthropometric variables, and HbA1c levels, suggesting no substantial baseline differences between participants who completed the intervention and those who withdrew.

### 3.3. Adherence

Table 3 presents adherence among the 12 participants in the INT who completed the 12-week remotely supervised exercise program. The mean total number of sessions completed was 19.4 ± 7.3, corresponding to an overall mean attendance rate of 64.7 ± 24.2% of the total prescribed sessions. The mean absolute weekly frequency was 1.6 ± 0.7 sessions per week throughout the intervention.

### 3.4. Clinical Outcomes

#### 3.4.1. Glycated Hemoglobin

HbA1c, the primary outcome of this study, showed no significant changes after the 12-week intervention (*p* = 0.30; *d* = −0.19, trivial). The results are presented in Table 4. Given that this result may have been underpowered to detect clinically relevant changes due to the small sample size, a descriptive subgroup analysis within the INT by baseline HbA1c values was conducted. It showed that participants with HbA1c ≥ 7.0% demonstrated greater absolute reductions over time (from 9.00% to 8.50%), whereas those with HbA1c < 7.0% remained relatively stable (from 6.04% to 6.21%) (Supplementary Table S2). Additionally, an exploratory descriptive subgroup analysis within the INT by adherence level revealed heterogeneous responses among individuals. Participants with low adherence (<70% of prescribed sessions attended) completed an average of 10.5 sessions and exhibited a 0.57% reduction in HbA1c, whereas those with high adherence (≥70%) completed an average of 24 sessions and showed minimal change (+0.08%) (Supplementary Table S3). These findings should be interpreted cautiously, given the exploratory nature of the analyses and the small subgroup sample sizes.

#### 3.4.2. Blood Pressure

No significant changes were observed for SBP (*p* = 0.51; *d* = −0.07, trivial) or DBP (*p* = 0.87; *d* = 0.07, trivial) after the 12-week intervention. The results are also presented in Table 4.

### 3.5. Functional Outcomes

The results for functional variables are presented in Table 5. After 12 weeks, significant group × time interactions were observed for the 30 s Chair Stand test (*p* = 0.02; *d* = 0.15, trivial), Arm Curl (*p* < 0.001; *d* = 1.24, large), TUG at usual speed (*p* = 0.01; *d* = −0.26, small), and the 2-Minute Step Test (*p* = 0.01; *d* = 1.32, large), indicating greater improvements from pre- to post-intervention in the INT compared with the CON. No significant interactions were observed for TUG at maximal speed (*p* = 0.11; *d* = −0.26, small) or the sit-and-reach test (*p* = 0.14; *d* = 0.12, trivial).

### 3.6. Psychosocial Outcomes

Table 6 presents the results of the psychosocial outcomes. Higher scores indicate better QOL, whereas lower scores indicate better outcomes for SQ, DS, and ED. For SQ, a significant group × time interaction was observed (*p* < 0.001), with a moderate effect size (*d* = −0.67). The INT showed a significant reduction in the mean SQ score, whereas the CON showed no significant change over time.

For DS, the analysis revealed a significant main effect of time (*p* = 0.03), with a small effect size (*d* = −0.30), and no significant group × time interaction (*p* = 0.19), indicating a similar reduction in both groups.

No significant interactions were observed for QOL (*p* = 0.37; *d* = 0.21, small) and ED (*p* = 0.08; *d* = −0.35, small).

### 3.7. Intervention-Only Outcomes

Data on capillary blood glucose values at pre- and post-session time points in mesocycles 1, 2, and 4 for the completers are presented in Table 7. A significant reduction in blood glucose levels was observed after the exercise session in all analyzed mesocycles (*p* = 0.03), with small effect sizes in mesocycles 1 (*d* = −0.24) and 2 (*d* = −0.27), and a trivial effect size in mesocycle 4 (*d* = −0.13).

At the end of the 12-week intervention, participants who completed the study reported a high level of satisfaction with their participation: 10 participants indicated the highest level on the scale (“very satisfied”), while 1 indicated “satisfied.” One participant did not report their level of satisfaction.

### 3.8. Other Outcomes

Eating habits and physical activity levels are presented in Table 8 and Table 9. No significant changes over time or between-group differences were observed for the consumption of in natura/minimally processed foods, processed/ultra-processed foods, overall eating habits score, or physical activity levels.

## 4. Discussion

The results of this study demonstrated that a synchronous telehealth exercise program did not affect HbA1c levels in individuals with T2D; however, it produced superior positive effects compared with the CON on functional fitness outcomes (30 s Chair Stand, Arm Curl, TUG at usual speed, and 2-Minute Step Test) and SQ, while DS decreased in both groups. In addition, the program exhibited an acute reduction in blood glucose across mesocycles 1, 2, and 4. These findings were observed in participants with a mean age of 56 years, a body mass index of 34 kg/m^2^ (class I obesity) [38], a mean HbA1c of 7.0%, and a mean disease duration of 8 years.

It is important to note that HbA1c was the primary outcome of the present study, and the sample size was calculated specifically to detect changes in this variable. Therefore, analyses involving secondary outcomes should be interpreted as exploratory, since no formal correction for multiple comparisons was applied.

An acute reduction in blood glucose levels was observed immediately after sessions in mesocycles 1, 2, and 4 among participants who completed the intervention, suggesting that the intervention produced an immediate glycemic effect in these individuals. To the best of our knowledge, this is the first study to systematically evaluate these acute responses throughout a structured remotely supervised exercise program. This effect may be explained by increased glucose uptake mediated by muscle contraction through pathways that are partially independent of insulin [1,6,39]. However, because these results were derived only from participants who completed the intervention, they should be interpreted with caution and may be subject to selection bias.

However, despite the acute response, HbA1c levels were not significantly affected after the 12-week intervention period. These findings should be interpreted with caution, since the final sample size was smaller than originally estimated and participant dropout in the INT may have reduced the statistical power to detect clinically meaningful between-group differences. Furthermore, the confidence interval was relatively wide and still included potentially clinically relevant effects favoring the intervention (up to −0.86%). Therefore, the findings do not support concluding that the intervention was ineffective; rather, they suggest imprecision in the estimated effect, possibly related to the limited sample size.

In addition, participants already presented HbA1c values close to the recommended target (<7%) [40], indicating relatively adequate metabolic control at baseline. This characteristic may have limited the potential for clinically meaningful improvements over time, suggesting a possible floor effect. Supporting this interpretation, a supplementary descriptive subgroup analysis showed that participants with baseline HbA1c values ≥ 7.0% demonstrated greater reductions following the intervention, whereas those already below the recommended target showed minimal changes. Although exploratory, these findings suggest greater benefits among individuals with poorer baseline glycemic control. Furthermore, reduced adherence across mesocycles resulted in a lower exercise volume effectively performed throughout the intervention. Although most participants achieved high adherence (≥70% of prescribed sessions attended), exploratory subgroup analyses according to adherence level showed no clear pattern between adherence and HbA1c changes (Supplementary Table S3). Given the small subgroup sample sizes, these findings should be interpreted with caution. Considering that reductions in HbA1c are generally associated with greater exercise volume [41,42], no conclusions regarding a dose–response relationship can be drawn from these exploratory analyses.

Despite this, the synchronous telehealth exercise program significantly improved the functional fitness in individuals with T2D. These findings may reflect neuromuscular adaptations induced by training, suggesting attenuation of functional deficits associated with the pathophysiology of T2D, such as loss of motor units, reduced rate of force development, and impaired motor control, which contribute to poorer functional performance and increased risk of falls [2]. Furthermore, physical performance measures, such as usual gait speed, are associated with clinically relevant outcomes, including increased survival in older adults [43]. Improvements in TUG at usual speed and in the 2-Minute Step Test indicate enhanced locomotor capacity, a variable recognized as a prognostic marker of overall health.

Overall, the functional performance outcomes should be interpreted in light of multiple interacting factors. Improvements observed in the intervention group across several tests are consistent with the potential beneficial effects of the supervised synchronous exercise program on neuromuscular function and functional capacity. However, these findings highlight the exploratory nature of functional outcomes in this trial and support cautious interpretation regarding clinical significance.

Although a statistically significant improvement was observed in the 30 s Chair Stand Test, the effect size was trivial, suggesting limited clinical relevance and warranting cautious interpretation of this outcome. Similarly, the improvement observed in the Arm Curl test in the INT, together with the decline observed in the CON, should be interpreted with caution, given the exploratory nature of the functional outcomes in this trial. Despite the small magnitude of differences observed in some functional outcomes and the inherent variability of these tests, even modest functional gains may be clinically meaningful in individuals with T2D, who commonly present with reduced functional capacity, greater disability, and higher levels of frailty compared with non-diabetic peers [44,45,46].

The improvement observed in the 30 s Chair Stand Test (1.5 repetitions) was smaller than that previously reported in the literature (4.66 repetitions) [47], possibly due to the low complexity and pragmatic nature of the intervention. Nevertheless, the entirely positive confidence interval for the between-group differences (0.23 to 2.73), together with the methodological rigor of the study, including randomization, standardized assessment procedures, and assessor blinding, reduces the likelihood that the observed changes are attributable solely to measurement variability.

Similarly, considering the characteristics commonly observed in individuals with T2D, including greater frailty [44,45,46], functional disability [44,45,46], reduced rate of force development [2] and loss of motor units [2], the improvement observed in the Arm Curl test in the INT, together with the decline observed in the CON, strengthens the likelihood of a true intervention effect rather than random variability.

Recommendations from the International Conference on Frailty and Sarcopenia Research emphasize that muscle strength and mobility are central determinants of functional independence and recommend resistance training as a priority strategy, even in the presence of chronic diseases [48]. Our findings are consistent with a systematic review and meta-analysis [47] demonstrating a robust association between exercise interventions and improvements in functional capacity in individuals with T2D. These improvements reinforce the role of physical exercise in preserving mobility and functional independence in this population, while also expanding the literature by demonstrating that such benefits can be achieved through remote supervision.

The study also demonstrated a significant improvement in SQ in the INT. This finding may be partially explained by physiological mechanisms proposed in the previous literature. Aerobic exercise has been associated with increased release of serotonin, norepinephrine, and melatonin, neurotransmitters involved in sleep–wake regulation, as well as reductions in REM sleep latency and wake time [49]. Resistance exercise has also been suggested to stimulate brain-derived neurotrophic factor (BDNF), which may contribute to neural plasticity and the regulation of neural circuits associated with sleep [49]. In addition, both exercise modalities have been associated with autonomic nervous system modulation and reduced cortisol levels, which may contribute to improved stress regulation and more restorative sleep [49]. However, these mechanisms were not directly assessed in the present study and should be interpreted as hypothetical.

The literature shows that individuals with T2D frequently present sleep disturbances, such as insomnia and sleep apnea, conditions associated with poorer glycemic control and a higher risk of cardiovascular complications [7]. Therefore, reductions in the PSQI score, corresponding to improvements in SQ, have important clinical implications for disease management. Previous studies have also demonstrated the benefits of traditional and mind–body exercise on subjective sleep quality in adults [50] and older adults [51]. A systematic review and meta-analysis by Rias et al. (2024) [52] reinforces this evidence in individuals with T2D and highlights the need for clinical trials with greater methodological rigor. To date, no studies with protocols similar to the present study have been identified, making our findings particularly relevant.

Regarding QOL, no significant effects were observed. Different results were reported by Terkes et al. (2023) [15], who found significant improvements in QOL in both groups, with superiority of the INT after six weeks of remote training (60 min, 3×/week). Differences compared with our findings may be related to the greater training volume and session duration in that study, as well as to sample characteristics, including older participants and the use of an instrument specific to older adults (CASP-19), which limits direct comparisons. Similarly, Nambi et al. (2023) [53] reported significant improvements in QOL following an intervention (80 min, 4×/week) with up to 12 months of follow-up, whereas the CON received only usual care. The discrepancies relative to our study may be mainly explained by the higher training volume and weekly frequency of the intervention protocol.

Regarding mental health outcomes, a significant reduction in PHQ-9 score, corresponding to improvement in DS, was observed in both groups. This is a clinically relevant finding considering that approximately 20% of individuals with T2D present depression [4,5,6], a condition associated with poorer QOL [54]. On the other hand, no significant changes were identified in ED, suggesting that this outcome, which is more specific to the condition, may require targeted interventions.

It is important to highlight that the study was conducted during a period still impacted by the COVID-19 pandemic (recruitment between May 2022 and June 2023), with the official end of the international public health emergency occurring only in May 2023 [28]. Social restrictions, fear of infection, and prolonged uncertainty have been widely associated with worsening mental health indicators in the general population [55,56,57]. In this context, the pandemic is a shared temporal and contextual factor that may have influenced overall DS levels in both groups. Therefore, the reduction in DS and the maintenance of ED scores in both groups, in the absence of between-group differences, may be interpreted as favorable within this broader context, but should be interpreted with caution and not attributed to the intervention or the pandemic itself.

Physical exercise is widely recommended as a complementary or alternative approach to pharmacological and psychotherapeutic treatments in individuals with various comorbidities, including T2D [39,41,42,58]. Our study sought to address, in a remote context, relevant demands of this population, which often faces barriers to adherence to regular physical exercise [8,9], such as lack of time, comorbidities, and low motivation.

Despite this, adherence to the intervention represented one of the main challenges. It was observed that, even with an increase in weekly frequency, there was no corresponding increase in participant engagement (~1.6 sessions/week), particularly in the final mesocycles. This finding suggests that adjustments in training volume alone may not be sufficient to promote greater engagement. Accumulated fatigue in the post-pandemic context and intensive use of digital technologies may have contributed to reduced motivation over time. Despite synchronous video interaction, the telehealth modality may have limitations regarding participant engagement and perceived motivation.

The present study has several important strengths, including the implementation of a structured intervention based on progression principles and supervised by trained professionals, as well as the use of validated instruments to assess outcomes. It is also noteworthy for its high external validity, as the proposed model is accessible, low-cost, and applicable in a home-based setting, which is particularly relevant in a pandemic context. The inclusion of still underexplored outcomes, such as SQ and ED, further contributes to advancing knowledge in this field.

On the other hand, several limitations should be considered. Although the telehealth-based intervention increases accessibility, it also introduces methodological challenges, including subjective control of exercise intensity, the use of standardized alternative materials, and potential variability in adherence over time, which may have influenced the magnitude of the observed effects. In addition, the remote-monitoring context may have led to underestimation of exercise load, particularly during the final minutes of the free walking periods when the researcher was not directly supervising participants’ movement trajectories. In addition, physical activity behavior in the control group could not be objectively monitored and was assessed only through self-report, which may introduce measurement bias.

Furthermore, the greater participant-researcher interaction and supervision in the intervention group may have contributed to the observed outcomes beyond the exercise stimulus itself. Participant satisfaction findings should also be interpreted with caution, as these data were obtained only from participants who completed the intervention; those who withdrew did not complete the post-intervention assessment despite being invited. Similarly, adherence was described exclusively among completers, potentially overestimating the feasibility of the intervention. Furthermore, the study was conducted in a single center, which may limit the generalizability of the findings. The inclusion of participants with internet access and basic digital literacy may have introduced a more socioeconomically advantaged population. In addition, acute blood glucose responses were assessed only in participants who completed the intervention. Therefore, these results may be subject to selection bias.

Further limitations include the lack of strict control over meal intake and medication timing throughout the intervention, which may have influenced glycemic responses, and an imbalance in medication use between groups at baseline. Although this imbalance resulted from the randomization process, it may also have influenced the observed outcomes.

Finally, the study followed the prospectively registered protocol. However, some limitations should be acknowledged, including the failure to reach the estimated sample size and deviations from the planned statistical analysis approach, which included analysis of covariance (ANCOVA); linear mixed models were adopted instead due to their greater flexibility for repeated-measures analysis and better handling of missing data. Despite this modification, all analyses were conducted according to the intention-to-treat principle.

Thus, the findings reinforce synchronous telehealth as a potential strategy for individuals with T2D and highlight the need for future studies integrating physical, behavioral, and psychosocial aspects, particularly those related to adherence and intervention outcomes. Furthermore, the results expand the evidence base on accessible models of care, which is especially relevant in contexts where continuity of care and the adoption of healthy behaviors remain challenging.

## 5. Conclusions

The present study demonstrated that a synchronous telehealth exercise program was not associated with significant changes in HbA1c, the primary outcome of the study; however, these findings should be interpreted with caution due to the reduced final sample size and the adequate participants’ metabolic control at baseline. Moreover, the intervention was associated with improvements in functional fitness and SQ in individuals with T2D compared with the CON. In addition, a reduction in DS was observed at the end of the intervention in both groups, with no between-group differences. Overall, these findings suggest that remotely supervised synchronous exercise interventions may be a feasible strategy to promote functional and psychosocial health outcomes in individuals with T2D, although further studies with larger sample sizes are needed to confirm them.

## Figures and Tables

**Table 1:** Periodization of the 12-week synchronous telehealth exercise intervention.

Week	Block	Exercise	Sets	Duration	Interval Between Exercise	Intensity	Interval Between Sets
Week 1–3 2×/week	Block 1	Sit to stand; Wall push-up; Bilateral calf raises.	2	30 s	30 s	Usual movement speed	60 s
		Stationary marching		90 s		RPE 11–13	
	Block 2	Single-arm dumbbell row; Bridge; Abdominal crunch.	2	30 s	30 s	Usual movement speed	60 s
		Stationary marching.		90 s		RPE 11–13	
	Block 3	Free walking.	1	5 min	RPE 11–13		
Week 4–6 2×/week	Block 1	Half squat; single-arm bent-over row; bilateral calf raise.	3	30 s	30 s	Usual movement speed	60 s
		Stationary marching.		90 s		RPE 11–13	
		Free walking.	1	5 min	RPE 11–13		
	Block 2	Bridge; floor press; cross-body crunch.	3	30 s	30 s	Usual movement speed	60 s
		Stationary marching.		90 s		RPE 11–13	
	Block 3	Free walking.	1	5 min	RPE 11–13		
Week 7–9 3×/week	Block 1	Half squat; single-arm bent-over row; bilateral calf raise.	3	30 s	30 s	Usual movement speed	60 s
		Stationary marching.		90 s		RPE 11–13	
		Free walking.	1	5 min	RPE 11–13		
	Block 2	Bridge; floor press; cross-body crunch.	3	30 s	30 s	Usual movement speed	60 s
		Stationary marching.		90 s		RPE 11–13	
	Block 3	Free walking.	1	5 min	RPE 11–13		
Week 10–12 3×/week	Block 1	Half squat; bent-over two-arm dumbbell row; single-leg calf raise.	3	20 s	30 s	Maximal movement speed	60 s
		Stationary marching.		120 s		RPE 13–15	
		Free walking.	1	5 min	RPE 13–15		
	Block 2	Single-leg Bridge; single-arm floor press; bird dog.	3	20 s	30 s	Maximal movement speed	60 s
		Stationary marching.		120 s		RPE 13–15	
	Block 3	Free walking.	1	5 min	RPE 13–15		

Notes: RPE = Rating of Perceived Exertion.

**Table 2:** Descriptive characteristics of the participants are expressed as mean ± standard deviation or absolute frequency.

Variables	Intervention Group (*n* = 17)	Control Group (*n* = 16)	Total N (*n* = 33)
	**Mean ± SD**	**Mean ± SD**	**Mean ± SD**
Age (years)	56.9 ± 10.1	54.6 ± 10.3	55.8 ± 10.1
Height (cm)	163.5 ± 8.0	162.3 ± 11.1	162.9 ± 9.5
Body mass (kg)	89.8 ±18.0	93.0 ± 22.4	91.0 ± 20.0
BMI (kg/m^2^)	33.3 ± 6.3	35.0 ± 6.3	34.2 ± 6.2
Waist circumference (cm)	101.6 ± 12.0	104.3 ± 15.5	102.9 ± 13.6
WHtR	0.6 ± 0.8	0.6 ± 0.1	0.6 ± 0.1
HbA1c (%)	7.2 ± 1.9	6.9 ± 1.3	7.0 ± 1.6
Diabetes duration (years)	9.0 ± 7.6	7.0 ± 5.9	8.5 ± 6.7
	**Absolute** **Frequency (*n*)**	**Absolute** **Frequency (*n*)**	**Absolute** **Frequency (*n*)**
Sex			
Female	14	13	27
Male	3	3	6
Medical treatment			
Metformin	12	14	26
Sulfonylureas	6	4	10
DPP-4 Inhibitors	3	1	4
SGLT2 Inhibitors	1	1	2
Diuretics	5	11	16
Beta-Blockers	3	5	8
ACE Inhibitors	1	1	2
ARBs	6	7	13
Ca channel blockers	2	1	3
Statins	6	5	11
Antidepressants	5	4	9
Anxiolytics	2	1	3

*n* = sample size; BMI = body mass index; WHtR = waist-to-height ratio; HbA1c = glycated hemoglobin; DPP-4 = dipeptidyl peptidase-4; SGLT2 = sodium-glucose cotransporter-2; ACE = angiotensin-converting enzyme; ARBs = angiotensin receptor blockers; Ca = calcium.

**Table 3:** Total attendance, attendance by mesocycle, and weekly attendance in each mesocycle among participants who completed the intervention (mean ± standard deviation or absolute weekly frequency; *n* = 12).

	Mean ± SD	
Number of completed sessions (*n*)	19.4 ± 7.3	
Overall attendance (%)	64.7 ± 24.2	
	**Attendance (%)**	**Absolute Weekly Frequency (*n*)**
Mesocycle 1 attendance (%)	70.8 ± 33.4	1.4 ± 0.7
Mesocycle 2 attendance (%)	69.4 ± 30.0	1.4 ± 0.6
Mesocycle 3 attendance (%)	65.7 ± 25.3	2.0 ± 0.8
Mesocycle 4 attendance (%)	57.4 ± 28.7	1.7 ± 0.9

**Table 4:** Changes in glycated hemoglobin, systolic blood pressure, and diastolic blood pressure from baseline to 12 weeks in the intervention and control groups.

		Time			*p*-Value		
Variables	Groups (*n*)	Baseline Mean ± SD	Post-Intervention Mean ± SD	EMD [95% CI] INT − CON	Group	Time	Group × Time
HbA1c (%)	INT (*n* = 17)	7.1 ± 1.8	6.9 ± 1.6	−0.29 (−0.86 to 0.27)	0.70	0.98	0.30
	CON (*n* = 16)	6.8 ± 1.3	6.9 ± 1.4				
SBP (mmHg)	INT (*n* = 17)	127.5 ± 12.5	126.9 ± 14.0	−3.24 (−13.18 to 6.71)	0.73	0.65	0.51
	CON (*n* = 16)	124.3 ± 16.9	124.8 ± 13.8				
DBP (mmHg)	INT (*n* = 17)	74.8 ± 9.1	74.7 ± 6.9	−0.48 (−6.37 to 5.41)	0.80	0.52	0.87
	CON (*n* = 16)	75.3 ± 8.6	74.6 ± 10.5				

Linear mixed models using the intention-to-treat approach and post hoc analyses with estimated marginal means (LSD); *n* = sample size; INT = intervention group; CON = control group; SD = standard deviation; HbA1c = glycated hemoglobin; SBP = systolic blood pressure; DBP = diastolic blood pressure; EMD = estimated mean difference with 95% confidence interval (95% CI) between the intervention and control groups.

**Table 5:** Changes in functional tests from baseline to 12 weeks in the intervention and control groups.

		Time			*p*-Value		
Tests	Groups (*n*)	Baseline Mean ± SD	Post-Intervention Mean ± SD	EMD [95% CI] INT − CON	Group	Time	Group × Time
30 s Chair Stand (reps)	INT (*n* = 17)	12.2 ± 4.2	13.3 ± 2.6 †	1.48 (0.23 to 2.73)	0.11	<0.001	0.02
	CON (*n* = 16)	11.5 ± 2.4	11.6 ± 2.8				
Arm Curl (reps)	INT (*n* = 17)	15.1 ± 4.3	17.5 ± 4.3 †	4.65 (2.73 to 6.59)	0.23	0.67	<0.001
	CON (*n* = 16)	15.9 ± 3.0	13.7 ± 3.3				
TUG (max speed) (s)	INT (*n* = 17)	6.9 ± 1.5	6.9 ± 1.1	−0.66 (−1.46 to 0.15)	0.29	0.93	0.11
	CON (*n* = 16)	7.1 ± 1.7	7.5 ± 1.4				
TUG (usual speed) (s)	INT (*n* = 17)	8.9 ± 1.8	8.5 ± 1.1 †	−0.90 (−1.59 to −0.21)	0.49	0.03	0.01
	CON (*n* = 16)	8.9 ± 1.8	8.9 ± 2.0				
2 min Step Test (rep)	INT (*n* = 17)	53.8 ± 12.0	66.7 ± 28.7 †	18.05 (5.60 to 30.50)	0.014	0.24	0.01
	CON (*n* = 16)	48.8 ± 15.6	43.4 ± 12.6				
Sit-and-Reach (cm)	INT (*n* = 17)	12.9 ± 7.2	13.9 ± 8.0	2.11 (−0.76 to 4.99)	0.06	0.09	0.14
	CON (*n* = 16)	9.3 ± 6.5	9.5 ± 6.1				

Linear mixed models using the intention-to-treat approach and post hoc analyses with estimated marginal means (LSD); *n* = sample size; INT = intervention group; CON = control group; SD = standard deviation; reps = repetitions; s = seconds; TUG = Timed Up and Go; † indicates a significant favorable change for the intervention group considering a significant interaction; EMD = estimated mean difference with 95% confidence interval (95% CI) between intervention and control groups.

**Table 6:** Changes in quality of life, sleep quality, depressive symptoms, and diabetes-related emotional stress from baseline to 12 weeks in the intervention and control groups.

		Time			*p*-Value		
Variables (Points)	Groups (*n*)	Baseline Mean ± SD	Post-Intervention Mean ± SD	EMD [95% CI] INT − CON	Group	Time	Group × Time
QOL (8–40)	INT (*n* = 17)	26.7 ± 6.4	28.3 ± 5.8	1.38 (−1.73 to 4.50)	0.85	0.06	0.37
	CON (*n* = 16)	27.5 ± 4.9	27.9 ± 4.3				
SQ (0–21)	INT (*n* = 17)	9.8 ± 4.3	7.9 ± 3.0 †	−3.34 (−5.47 to −1.20)	0.53	0.24	<0.001
	CON (*n* = 16)	9.0 ± 4.7	10.2 ± 4.4				
DS (0–27)	INT (*n* = 17)	11.8 ± 7.8	8.4 ± 8.0	−2.62 (−6.64 to 1.40)	0.77	0.03	0.19
	CON (*n* = 16)	9.7 ± 8.0	8.8 ± 8.3				
ED (0–100)	INT (*n* = 17)	49.1 ± 32.3	45.0 ± 35.0	−12.98 (−27.59 to 1.62)	0.44	0.95	0.08
	CON (*n* = 16)	34.9 ± 27.0	41.1 ± 24.7				

Linear mixed models using the intention-to-treat approach and post hoc analyses with estimated marginal means (LSD); *n* = sample size; INT = intervention group; CON = control group; SD = standard deviation; QoL = quality of life; SQ = sleep quality; DS = depressive symptoms; ED = Emotional Distress; † indicates significant improvement for the intervention group considering a significant interaction; EMD = estimated mean difference with 95% confidence interval (95% CI) between intervention and control groups.

**Table 7:** Glycemic levels (mg/dL) before and immediately after a session at the initial period of mesocycles 1, 2, and 4 among participants who completed the intervention (*n* = 12).

	Time			*p*-Value		
Mesocycles	Pre-Session Mean ± SD	Post-Session Mean ± SD	EMD [95% CI] Pre − Post	Time	Mesocycle	Time × Mesocycle
1	164.4 ± 111.3	141.9 ± 71.8 *	−19.26 (−36.08 to −2.45)	0.03	0.73	0.87
2	164.9 ± 94.2	142.7 ± 70.8 *				
4	153.4 ± 114.9	140.3 ± 86.9 *				

Linear mixed models and post hoc analyses using estimated marginal means (LSD); *n* = sample size; SD = standard deviation; * indicates significant difference between pre- and post-session values for the INT in each mesocycle; EMD = estimated mean difference between pre- and post-exercise time points with 95% confidence interval (95% CI).

**Table 8:** Changes in dietary intake from baseline to 12 weeks in the intervention and control groups.

		Time			*p*-Value		
Variables	Groups (*n*)	Baseline Mean ± SD	Post-Intervention Mean ± SD	EMD [95% CI] INT − CON	Group	Time	Group × Time
In natura/minimally processed foods	INT (*n* = 17)	16.2 ± 2.7	15.7 ± 2.6	−0.85 (−2.81 to 1.10)	0.17	0.91	0.38
	CON (*n* = 16)	17.0 ± 2.6	17.4 ± 2.9				
Processed/ultra-processed foods	INT (*n* = 17)	23.6 ± 5.0	23.5 ± 5.6	0.83 (−2.35 to 4.02)	0.10	0.71	0.60
	CON (*n* = 16)	20.4 ± 7.2	20.3 ± 7.8				
Overall score	INT (*n* = 17)	39.9 ± 5.6	39.3 ± 5.9	−0.08 (−3.49 to 3.34)	0.23	0.80	0.97
	CON (*n* = 16)	37.4 ± 6.5	37.7 ± 6.1				

Linear mixed models using the intention-to-treat approach and post hoc analyses with estimated marginal means (LSD); *n* = sample size; INT = intervention group; CON = control group; SD = standard deviation; EMD = estimated mean difference with 95% confidence interval (95% CI) between intervention and control groups.

**Table 9:** Classification of participants according to physical activity level (IPAQ-C) at baseline and post-intervention. with within- and between-group comparisons.

Group	Classification	Baseline	Post-Intervention	*p*-Value
Intervention group (*n* = 17)	Very active	2	4	0.75 ^b^
	Active	8	7	
	Insufficiently active	7	1	
Control group (*n* = 16)	Very active	4	3	0.60 ^b^
	Active	4	10	
	Insufficiently active	8	3	
	*p*-value	0.36 ^a^	0.57 ^a^	

^a^ Chi-square test between groups for each time point; ^b^ exact symmetry test between pre- and post-intervention moments for each group.

## Data Availability

The datasets generated and/or analyzed during the current study are available from the corresponding author upon reasonable request.

## Supplementary Materials

The following supporting information can be downloaded at: https://www.mdpi.com/article/10.3390/ijerph23060773/s1, Table S1. Baseline characteristics of completers and non-completers within the intervention group. Table S2. Descriptive subgroup analysis according to baseline HbA1c values for the completer participants within the intervention group (*n* = 12). Table S3. Exploratory descriptive subgroup analysis of HbA1c according to adherence level among intervention completers (*n* = 12).

## Abbreviations

The following abbreviations are used in this manuscript:

T2D	Type 2 diabetes mellitus
INT	Intervention group
CON	Control group
HbA1c	Glycated hemoglobin
ED	Emotional distress
BHUs	Basic Health Units
SUS	Unified Health System
RPE	Rating of Perceived Exertion
BP	Blood pressure
QOL	Quality of life
SQ	Sleep quality
DS	Depressive symptoms
HPLC	High-performance liquid chromatography
SBP	Systolic blood pressure
DBP	Diastolic blood pressure
TUG	Timed Up And Go
PSQI	Pittsburgh Sleep Quality Index
PHQ-9	Patient Health Questionnaire-9
B-PAID	Brazilian version of the problem areas in diabetes
SD	Standard deviation
BMI	Body mass index
IPAQ	International Physical Activity Questionnaire
METs	Metabolic equivalents
FFQ	Food frequency questionnaire
95% CI	95% confidence interval
ITT	Intention-to-treat
BDNF	Brain-derived neurotrophic factor
