# Supplementary material for: Effects of a Synchronous Telehealth Exercise Program on Clinical, Functional, and Psychosocial Outcomes in Individuals with Type 2 Diabetes Mellitus (RED Study): A Randomized Clinical Trial
Source: Int J Environ Res Public Health. 2026 Jun 8;23(6):773. doi: 10.3390/ijerph23060773 (PMC13299911; doi:10.3390/ijerph23060773)
Supplement: Supplementary file 1 [file ijerph-23-00773-s001.zip › ijerph-4344348-supplementary.pdf]

Supplementary Material

Manuscript Title: Effects of a Synchronous Telehealth Exercise Program on Clinical, Functional, and Psychosocial Outcomes in Individuals with Type 2 Diabetes Mellitus (RED Study): A randomized clinical trial

Samara Nickel Rodrigues [1], Bruno Veiga Guterres [1], Maurício Tatsch Ximenes Carvalho [1], Rodrigo Sudatti Delevatti [2] and Cristine Lima Alberton [1,*]

[1] School of Physical Education and Physiotherapy, Federal University of Pelotas, Pelotas 96055-630, RS, Brazil; samara-nrodrigues@hotmail.com (S.N.R.); bveigaguterres@gmail.com (B.V.G.); carvalhomauricio960@gmail.com (M.T.X.C.)
[2] Center of Sports, Department of Physical Education, Federal University of Santa Catarina, Florianopolis 88040-900, SC, Brazil; rodrigo.delevatti@ufsc.br
* Correspondence: cristine.alberton@ufpel.edu.br

Journal: International Journal of Environmental Research and Public Health

Summary of Supplementary Material

- **Supplementary Results**

Supplementary Results

Supplementary Table S1. Baseline characteristics of completers and non-completers within the intervention group.

Variables	Completers (*n* = 12)	Non-completers (*n* = 5)
	Mean ± SD	Mean ± SD
Sex		
Female	10	4
Male	2	1
Age (years)	55.6 ±8.9	57.7 ± 13.0
Height (cm)	165.2 ± 6.9	160.5 ± 9.7
Body mass (Kg)	88.8 ±16.4	89.8 ± 22.3
BMI (Kg/m^2)	32.5 ± 5.1	34.9 ± 8.4
Waist circumference (cm)	100.7 ± 12.7	103.2 ± 11.6
WHtR	0.6 ± 0.1	0.6 ± 0.1
HbA1c (%)	7.1 ± 2.0	7.2 ± 1.6
Diabetes duration (years)	9.0 ± 8.3	9.8 ± 7.3

n = sample size; BMI = body mass index; WHtR = waist-to-height ratio; HbA1c = glycated hemoglobin.

Supplementary Table S2. Descriptive subgroup analysis according to baseline HbA1c values for the completer participants within the intervention group (*n* = 12).

**Baseline HbA1c**	*n*	Pre	Post	Δ
< 7.0%	8	6.04	6.21	+ 0.17
≥ 7.0%	4	9.00	8.50	- 0.50

Only participants with complete pre- and post-intervention HbA1c data were included in this descriptive analysis; Values are presented as mean HbA1c (%) at baseline (Pre) and post-intervention (Post); (Δ) represents the absolute change in HbA1c from baseline to post-intervention.

Supplementary table S3. Exploratory descriptive subgroup analysis of HbA1c according to adherence level among intervention completers (n = 12).

Adherence level	n	Mean sessions completed	**HbA1c (%)**		
			Pre	Post	**Δ**
< 70%	4	10.5	7.00	6.43	- 0.57
≥ 70%	8	24.0	7.11	7.19	+ 0.08

Adherence was categorized as high (≥ 70% of prescribed sessions attended) or low (< 70% of prescribed sessions attended). Values are presented as mean HbA1c (%) at baseline (Pre) and post-intervention (Post), with the corresponding within-group change (Δ). Only participants with complete pre- and post-intervention HbA1c data were included in this exploratory analysis.
